# Frequency and variability of nonmetric dental crown traits of primary and permanent molars in a group of orthodontic patients

**DOI:** 10.1007/s00056-024-00532-3

**Published:** 2024-06-06

**Authors:** Ariane Beatriz Blancato, Eva Paddenberg-Schubert, Peter Proff, Maria Angélica Hueb de Menezes-Oliveira, Svenja Beisel-Memmert, Flares Baratto-Filho, Carsten Lippold, Christian Kirschneck, Erika Calvano Küchler, Cesar Penazzo Lepri

**Affiliations:** 1 Department of Biomaterials, University of Uberaba – UNIUBE, Uberaba, Minas Gerais Brazil; 2 Department of Orthodontics, University Medical Centre of Regensburg, Regensburg, Germany; 3 Department of Orthodontics, University of Bonn, Welschnonnenstr. 17, 53111 Bonn, Germany; 4 School of Dentistry, Tuiuti University from Paraná, Curitiba, Paraná Brazil; 5 Department of Dentistry, Univille – University from the Joinville Region, Joinville, Santa Catarina Brazil; 6 Private Orthodontic Practice, Synagogenstr. 1, Ibbenbüren, Germany

**Keywords:** Anatomy, Dentition, Tooth crown, Deciduous tooth, Orthodontics, Anatomie, Dentition, Zahnkrone, Dens deciduus, Kieferorthopädie

## Abstract

**Background:**

The present study aimed to assess the frequency and variation of 13 nonmetric dental crown traits (NDCT) in permanent and primary molars in German orthodontic patients.

**Methods:**

Dental records from orthodontic patients were screened and evaluated. First and second permanent and primary upper and lower molars (from left and right sides) were assessed. Teeth with cavitated dental caries, occlusal wear, restorations and obvious dental deformities were not evaluated. The NDCT for permanent molars were identified and scored according to the odontoscopic system developed by Arizona State University Dental Anthropology System (ASUDAS). The NDCT for primary molars were identified and scored according to ASUDAS, Hanihara’s method and Sciulli’s method. The χ^2^ test was used to investigate side preference and sexual dimorphism at a significance level of *p* ≤ 0.050.

**Results:**

A total of 163 orthodontic patients (82 males and 81 females) aged 8–14 years were included. A sexual dimorphism was observed for the hypocone in first upper permanent molar (*p* = 0.041). The protostylid was observed in lower permanent molars (range 2.1–10%). Males presented more hypoconulid than females (*p* = 0.019). Only females presented the distal trigonid crest in lower first permanent molars (*p* = 0.002). The most common groove pattern in primary molars was Y; male presented more Y grade than females in the lower second primary molar (*p* = 0.039). Asymmetry was observed in some traits, ranging from 0 to 100%.

**Conclusion:**

The present study showed the frequency of NDCT of molars in German orthodontic patients and demonstrated that some traits present sexual dimorphism.

**Supplementary Information:**

The online version of this article (10.1007/s00056-024-00532-3) contains supplementary material, which is available to authorized users.

## Abstract (de)

**Hintergrund:**

Ziel der vorliegenden Studie war es, die Variation und Häufigkeit 13 nicht metrischer Kronenmerkmale („nonmetric traits“, NDCT) von Milchmolaren und bleibenden Molaren innerhalb einer deutschen kieferorthopädischen Patientengruppe zu untersuchen.

**Material und Methoden:**

Anhand diagnostischer Patientenunterlagen wurden die ersten und zweiten Molaren jedes Quadranten der lactealen und permanenten Dentition untersucht. Zähne mit einer kavitierten Karies, Restauration, okklusalen Abnutzung oder offensichtlichen Malformation wurden nicht mit einbezogen. Die NDCT der bleibenden Molaren wurden nach dem odontoskopischen Arizona State University Dental Anthropology System (ASUDAS) bewertet. Die Analyse der NDCT von Milchmolaren erfolgte nach ASUDAS sowie nach der Hanihara- und der Sciulli-Methode. Seitenpräferenz sowie Geschlechtsdimorphismus wurden mittels χ^2^-Test auf einem Signifikanzniveau von *p* ≤ 0,050 ermittelt.

**Ergebnisse:**

Die Untersuchungsgruppe umfasste 163 kieferorthopädische Patienten (davon 82 männliche, 81 weibliche) im Alter zwischen 8 und 14 Jahren. Für den Hypokonus des ersten oberen bleibenden Molaren ergab sich ein Geschlechtsdimorphismus (*p* = 0,041). Das Protostylid wurde bei den unteren bleibenden Molaren identifiziert (zwischen 2,1 und 10 %). Das Hypoconulid konnte innerhalb der männlichen Untersuchungsgruppe häufiger nachgewiesen werden als in der weiblichen (*p* = 0,019). Ausschließlich weibliche Patienten wiesen einen distalen Trigonidkamm der unteren ersten bleibenden Molaren auf (*p* = 0,002). Das häufigste Fissurenmuster der Milchmolaren war Y‑förmig, bei den unteren zweiten Milchmolaren männlicher Patienten war es stärker ausgeprägt als bei weiblichen Patienten (*p* = 0,039). Einige Merkmale zeigten Asymmetrien von 0–100 %.

**Schlussfolgerung:**

Die Untersuchungen zeigen die Häufigkeit der NDCT von Milch- und permanenten Molaren in einer deutschen kieferorthopädischen Patientengruppe und deuten auf einen Geschlechtsdimorphismus einiger Merkmale hin.

## Introduction

Molars are the largest teeth in the oral cavity and due to their position and eruption time, a central pillars of the development of the occlusion. Human molars are found in the permanent and primary dentition and present a morphological variation that ranges in size and shape [1, 2]. The human dental morphology, particularly the molars morphology, has been described as presenting several parameters with a variety of morphological traits [3, 4]. The study of crown morphology, including identification of the molars’ traits, is based on evaluation of the occlusal surfaces [5]. Morphological categories have been used over the past century to describe nonmetric dental crown traits (NDCT) in occlusal surfaces of the mandibular and maxillary molars [6–9].

The first study of dental morphological characteristics in humans was reported by Hrdlička in 1920 [6]. This author described different expressions of a shovel shape on upper permanent incisors, in which the trait ranged from minimal to maximal expression [6, 9]. In 1956, Dahlberg [7] introduced scales with grades of trait expressions and a series of standardized plaques to study teeth’s morphological variations, such as Carabelli trait, protostylid and hypocone. In 1961, Hanihara [8] published an important study describing new classifications of morphological traits of the molars on primary dentition, as well as the crown pattern and distal trigonid crest. Turner et al. [9] developed a dental system for permanent molars called the ASUDAS (Arizona State University Dental Anthropology System) with an extensive series of crown and root trait classifications, which is currently the most widely used system for scoring dental morphology. The use of these systems in dental and anthropological research allows replicability among observers, and they produce data that express the variation tendency of all NDCT present on the molar crown [8, 10].

The analysis of dental morphology aims to explore the frequency, the existence of sexual dimorphism and bilateral symmetry of NDCT in permanent and primary teeth [11], evaluating the degrees of expression of these traits [12, 13] and allowing the comparison among different populations. However, dental morphology is not only of interest from an anthropological point of view as the shape of the occlusal relief also has an effect on the development of the dental arches. It has been shown in an animal model that interdigitation of molars and canines contributes to the development of the dental arches [14]. In a recent study, NDCT were associated with the development of different types of malocclusions in a human sample from India [15]. This highlights the importance to understand the frequency of different NDCTs in a wider sample of populations and how they are connected to the development of different malocclusions. There are few studies that investigated molars anatomy in humans [16–19], and the investigation of these traits in different populations are necessary [12, 13]. Therefore, this study aimed to investigate the frequency and variation of 13 NDCT in permanent and primary molars in German orthodontic patients. The presence of bilateral symmetry and sexual dimorphism was also investigated.

## Method

### Population and sampling

This descriptive cross-sectional study was approved by the institutional review board from the University of Regensburg (approval number ID: 19-1549-101). All included subjects and/or their legal guardians signed the informed consent prior to the inclusion in the study. Age-appropriate assent documents were also used for individuals younger than 14 years. This project was performed according to the Helsinki Declaration. This study was reported following the Statement of Strengthening the Reporting of Observational Studies in Epidemiology (STROBE) [20].

Dental casts (orthodontic diagnostic casts) from orthodontic patients (children older than 8 years old and teenagers) undergoing orthodontic treatment were consecutively selected by convenience at the University of Regensburg and private orthodontic practices in Regensburg, Germany. Dental cast from patients with syndromes, oral cleft, congenital alterations including tooth agenesis (except for third molar agenesis), and severe bruxism with dental tissue loss were excluded to prevent distortion of the data. To maximize data interpretability, only patients with a Middle-European ancestry (at maximum one grandparent not from Middle Europe) were included. Included individuals should have at least one set (maxilla and mandible) of dental casts. Some individuals presented more than one set of dental casts (at different ages and from different stages of the orthodontic treatment). In these cases, both dental casts were assessed in order to evaluate the majority of molars possible (primary and permanent), however, each tooth was evaluated only once.

### Morphological analysis

All dental casts were scanned and processed into the software using the three-dimensional (3D) measuring OrthoXScan 2.8 (Dentaurum, Ispringen, Germany). The software OnyxCeph^3^™ (version 3.2.52, Image Instruments GmbH, Chemnitz, Germany) was used to take images of the molars from the virtual 3D casts. First and second permanent and primary maxillary and mandibular molars (from left and right side) were assessed. Teeth with cavitated dental caries, occlusal wear, restorations and obvious dental deformities were not evaluated.

**Fig. 1:**
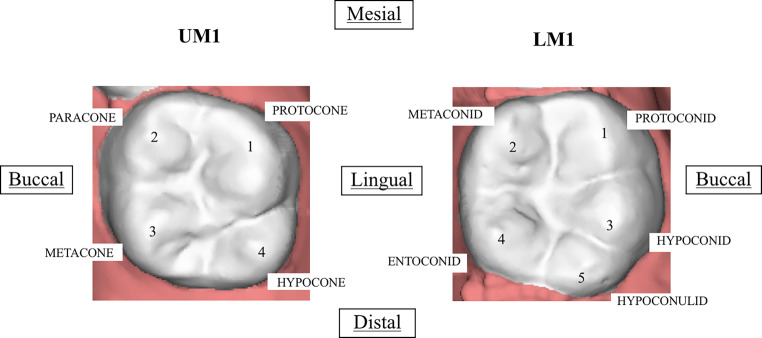
Permanent maxillary and mandibular right first molar showing cusps indicated by numbers according to their location. *UM1* permanent upper molar 1, *LM1* permanent lower molar 1 Bleibender oberer und unterer rechter erster Molar. Die Höcker sind entsprechend ihrer Lage nummeriert. *UM1* bleibender oberer Molar 1, *LM1* bleibender unterer Molar 1

The NDCT for permanent molars were identified and scored according to the odontoscopic system developed from Arizona State University Dental Anthropology System (ASUDAS) [9]. The ASUDAS uses standard recording forms to evaluate traits and the variability of their expressions, which are shown in Table 1 and illustrated in Fig. 1. For the hypocone trait, grades 1 and 2, and grades 3 and 4 were grouped and assessed as having the same degree of expression. The name and position of each cusp are also illustrated in Fig. 1.

**Table 1:** Classification used to assess the morphology of the permanent upper and lower molars Klassifikation zur Beurteilung der Morphologie der oberen und unteren bleibenden Molaren

Trait	Features	Grade
Carabelli’s trait^(UM)^	It occurs on the lingual surface of the protocone (mesiolingual cusp) and is expressed from complete absence, groove or pit (regarded a negative expression) to a large cusp (positive expression)	*0.* Smooth mesiolingual surface
		*1.* Vertical groove present
		*2.* Pit present
		*3.* Small Y‑shaped depression
		*4.* Large Y‑shaped depression
		*5.* Small cusp without free apex
		*6.* Medium cusp with free apex making contact with the medial lingual groove
		*7.* Large free cusp
Metacone^(UM)^	The upper molar has 3 major main cusps regarding the metacone cusp. It is named the distobuccal cusp or cusp 3	*0.* Absent
		*1.* There is a ridge but no free apex
		*2.* Faint cuspule with a free apex
		*3.* Weak cusp
		*3.5.* Intermediate-sized cusp that falls between grades 3 and 4
		*4.* Large cusp
		*5.* Very large cusp, equal in size to a large hypocone
Hypocone^(UM)^	The distolingual cusp is derived from the cingulum and attached to the distolingual surface of the trigon (protocone or cusp 1, paracone or cusp 2 and metacone or cusp 3)	*0.* Smooth surface
		*1.* Faint ridge
		*2.* Faint cuspule
		*3.* Small cusp
		*3.5.* Moderate-sized cusp
		*4.* Large cusp
		*5.* Very large cusp
Metaconule^(UM)^	A 5th cusp on the distal border between the metacone (cusp 3) and hypocone (cusp 4). The cusp shows two parallel vertical grooves	*0.* Trait is absent
		*1.* Faint cuspule
		*2.* Trace cuspule
		*3.* Small cuspule
		*4.* Small cusp;
		*5.* Medium-sized cusp
Parastyle^(UM)^	It is most commonly expressed on the paracone (mesiobuccal cusp or cusp 2). In some instances, it occurs on the metacone (cusp 3)	*0.* Smooth buccal surface
		*1.* A small pit near the buccal groove
		*2.* Small cusp without free apex
		*3.* Medium cusp with free apex
		*4.* Large cusp with free apex
		*5.* Very large cusp that may extend onto the surfaces of both cusps 2 and 3
		*6.* Peg-shaped crown attached to root of second or third molar
Groove pattern^(LM)^	Lower molars can have 5 cusps: cusp 1 (protoconid), cusp 2 (metaconid), cusp 3 (hypoconid), cusp 4 (entoconid) and cusp 5 (hypoconulid). The cusps can make contacts creating patterns	*Y.* Cusps 2 and 3 are in contact
		*+.* Cusps 1–4 are in contact
		*X.* Cusps 1 and 4 are in contact
Hypoconulid^(LM)^	The cusp 5 occurs on the distal occlusal surface and it associated with cusp 3 (hypoconid)	*0.* Absent. The molar has only 4 cusps
		*1.* Very small
		*2.* Small
		*3.* Medium-sized
		*4.* Large
		*5.* Very large
Cusp 6^(LM)^	This cusp is expressed on the distal portion, but it is associated with the cusp 4 (entoconid). It is important to note that cusp 5 (hypoconulid) has to be present	*0.* Absent
		*1.* Much smaller than cusp 5
		*2.* Smaller than cusp 5
		*3.* Equal in size to cusp 5
		*4.* Large than cusp 5
		*5.* Much larger than cusp 5
Cusp 7^(LM)^	It is a wedge-shaped accessory cusp expressed in the lingual groove between cusps 2 (metaconid) and 4 (entoconid). Cusp 7 is never considered in determining cusp number	*0.* Absent
		*1.* Faint cusp, two weak lingual grooves
		*1A.* Faint tipless displaced on the lingual surface of cusp 2
		*2.* Small
		*3.* Medium-sized
		*4.* Large
Protostylid^(LM)^	This trait is a cingular derivate on the buccal surface associated with the buccal groove, particularly separating cusp 1 (protoconid) and cusp 3 (hypoconid)	*0.* Smooth surface
		*1.* Pit present
		*2.* Buccal groove curve distal
		*3.* Faint groove extending mesial from the buccal groove
		*4.* Groove more pronounced
		*5.* Groove stronger
		*6.* Groove extend across the buccal surface
		*7.* Free cusp
Anterior Fovea^(LM)^	This trait is expressed on the mesial occlusal surface. It involves distinct essential ridges on cusp 1 (protoconid) and cusp 2 (metaconid) that meet close to the centre of the trigonid, and a mesial marginal ridge that is expressed to varying degrees	*0.* Absent
		*1.* Trace, with a weak ridge connecting the mesial aspects
		*2.* Essential ridges on trigonid better developed and resulting groove deeper than in grade 1
		*3.* Essential ridges pronounced and marginal ridge well developed, producing a distinctive fovea on the anterior portion of the trigonid
		*4.* The mesial ridge is robust and the marginal ridge produces a well-defined fovea with a very long groove
Deflecting Wrinkle^(LM)^	The form of manifestation of the essential medial ridge on cusp 2 (metaconid)	*0.* Absent
		*1.* The essential ridge is straight and shows a midpoint constriction
		*2.* The essential ridge is deflected distally. There is no contact with cusp 4 (entoconid)
		*3.* The essential ridge shows strong deflection at the midpoint forming an L‑shaped ridge. There is contact with cusp 4 (entoconid)
Distal Trigonid Crest^(LM)^	The major mesial cusps of the trigonid (protoconid or cusps 1, and metaconid or cusp 2) express distal accessory ridges that are directly connected along the distal portion of the cusps. They can be continuous or discontinuous	*0.* Absent
		*1.* Present: distal borders are connected by a ridge

ASUDAS method was used [9]

^(UM)^permanent upper molar, ^(LM)^permanent lower molar

**Fig. 2:**
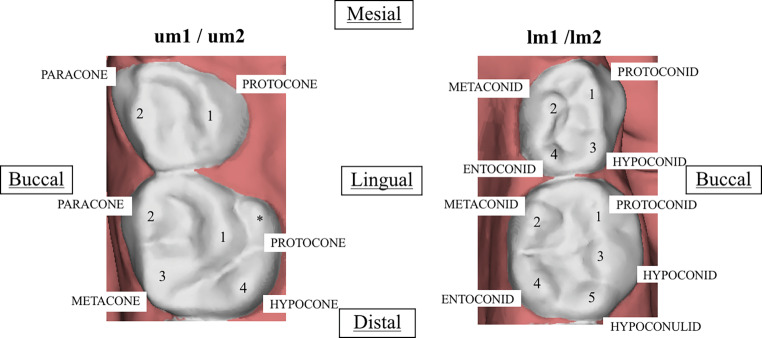
Primary maxillary and mandibular right first and second molars showing cusps indicated by numbers according to their location. *um1/um2* primary upper molar 1/2, *lm1/lm2* primary lower molar 1/2, *asterisk* Carabelli’s trait Erste und zweite Milchmolaren des Ober- und Unterkiefers rechts. Die Höcker sind entsprechend ihrer Lage nummeriert. *um1/um2* lactealer oberer Molar 1/2, *lm1/lm2* lactealer unterer Molar 1/2, *Asterisk* Carabelli-Zeichen

The NDCT for primary molars were identified and scored according to the ASUDAS [9], Hanihara [8] and Sciulli methods [21]. The evaluated traits and the variability of their expressions are shown in Table 2 and illustrated in Fig. 2. The name and position of each cusp are illustrated in Fig. 2.

**Table 2:** Classification used to assess the morphology of the primary upper and lower molars Klassifizierung zur Beurteilung der Morphologie der primären oberen und unteren Molaren

Trait	Features	Grade
Carabelli’s trait^3 (um)^	It occurs on the lingual surface of the protocone (mesiolingual cusp). This tuberculum projection is expressed from complete absence, groove or pit to a large cusp	*0.* Smooth surface
		*1.* Pit, groove
		*2.* Two grooves are roughly parallel
		*3.* The area between grooves raised, apex not free
		*4.* The expression is similar to type 3 but there is a free apex
Crown pattern 1st molar^2 (um)^	The pattern is based on the development of the crown cusps of the upper 1st deciduous molars	*2.* Protocone and paracone
		*3M.* Protocone, paracone and metacone
		*3H.* Protocone, paracone and hypocone
		*4−.* All four cusps but hypocone reduced
		*4.* All four cusps but hypocone not reduce
Crown pattern 2nd molar^2 (um)^	The pattern is based on the development of the crown cusps of the upper 2nd deciduous molars	*3.* Protocone, paracone, metacone, and a small hypocone. The distal marginal ridge can be interrupted by a groove
		*4−.* Protocone, paracone, metacone, and a small hypocone. The distal marginal ridge follows its course without interruption
		*4.* Protocone, paracone, metacone, and large hypocone
Metaconule^3 (um)^	A 5th cusp on the distal border between the metacone and hypocone. The cusp shows two parallel vertical grooves	*0.* Absent
		*P.* Present
Parastyle^1 (um)^	It is most common expressed on the paracone (mesiobuccal cusp). In some instances, it occurs on the metacone	*0.* Smooth buccal surface
		*1.* A small pit near the buccal groove
		*2.* Small cusp without free apex
		*3.* Medium cusp with free apex
		*4.* Large cusp with free apex
		*5.* Very large cusp that may extend onto the surfaces of both cusps 2 (paracone) and 3 (metacone)
		*6.* Peg-shaped crown attached to root of second or third molar
Groove pattern^1 (lm)^	Lower molars can have 5 cusps: cusp 1 (protoconid), cusp 2 (metaconid), cusp 3 (hypoconid), cusp 4 (entoconid) and cusp 5 (hypoconulid). The cusps can make contacts creating patterns	*Y.* Cusps 2 and 3 are in contact
		*+.* Cusps 1–4 are in contact
		*X.* Cusps 1 and 4 are in contact
Hypoconulid^3 (lm)^	Cusp 5 occurs on the distal occlusal surface and is associated with cusp 3 (hypoconid)	*0.* Absent
		*P.* Present
Cusp 6^1 (lm)^	This cusp is expressed on the distal portion, but it is associated with the cusp 4 (entoconid). It is important to note that cusp 5 (hypoconulid) has to be present	*0.* Absent
		*1.* Much smaller than cusp 5
		*2.* Smaller than cusp 5
		*3.* Equal in size to cusp 5
		*4.* Large than cusp 5
		*5.* Much larger than cusp 5
Cusp 7^2 (lm)^	It is a wedge-shaped accessory cusp expressed in the lingual groove between cusps 2 (metaconid) and 4 (entoconid). Cusp 7 is never considered in determining cusp number	*0.* Absent
		*1.* Only a very weak short groove extends downward from the lingual ridge of cusp 3 (hypoconid). There is no cusp
		*2.* There are two grooves on the lingual surface and a small cusp is present
		*3.* The cusp is well developed
Protostylid^1 (lm)^	This trait is a cingular derivate on the buccal surface associated with the buccal groove, particularly separating cusp 1(protoconid) and cusp 3 (hypoconid)	*0.* Smooth surface
		*1.* Pit present
		*2.* Buccal groove curve distal
		*3.* Faint groove extending mesial from the buccal groove
		*4.* Groove more pronounced
		*5.* Groove stronger
		*6.* Groove extend across the buccal surface
		*7.* Free cusp
Anterior fovea^1 (lm)^	This trait is expressed on the mesial occlusal surface. It involves distinct essential ridges on cusp 1 (protoconid) and cusp 2 (metaconid) that meet close to the centre of the trigonid, and a mesial marginal ridge that is expressed to varying degrees	*0.* Absent
		*1.* Trace, with a weak ridge connecting the mesial aspects
		*2.* Essential ridges on trigonid better developed and resulting groove deeper than in grade 1
		*3.* Essential ridges pronounced and marginal ridge well developed, producing a distinctive fovea on the anterior portion of the trigonid
		*4.* The Mesial ridge is robust and the marginal ridge produces a well-defined fovea with a very long groove
Central ridge of the metaconid^2 (lm)^	The form of manifestation of the essential medial ridge on cusp 2 (metaconid). This trait is the same as Deflecting Wrinkle on permanent dentition	*1.* The essential ridge of the cusp 2 is expressed similar in size and prominence as that of the other cusps
		*2.* The essential ridge is very well developed in its thickness on cusp 2 and also expands its width in the trigonid basin. The ridge sometimes seems to curve distally at its inner end
Distal trigonid crest^2 (lm)^	The major mesial cusps of the trigonid (cusps 1 and 2) express distal accessory ridges that are directly connected along the distal portion of the cusps. They can be continuous or discontinuous	*0.* Absent
		*1.* Present: distal borders are connected by a ridge

^1^ASUDAS methods [9], ^2^Hanihara methods (1961), ^3^Sciulli methods (1998), ^(um)^deciduous upper molar, ^(lm)^deciduous lower molar

Examples of NDCT for primary molars (Supplementary Fig. 1) and for permanent molars (Supplementary Fig. 2) are shown in the supplementary figures.

To describe the jaw, letters were used: UM for permanent upper molar, um for primary upper molar, and LM for permanent lower molar, lm for a primary lower molar. To describe the tooth position, numbers were used: 1 for the first molar, and 2 for the second molar [22].

All analyses were performed by one single trained and calibrated examiner. The kappa (κ) test for intraobserver reliability was performed, in which the assessments were carried out twice by the same examiner within a 2-week interval. The κ values for agreement ranged from 0.71 to 1.00.

### Statistical analysis

The prevalence of each trait as well as each variability expression were described. Left–right symmetry/asymmetry were evaluated only in subjects that presented the molar and its contralateral available for analysis. Also, symmetry and asymmetry were only investigated when the trait (regardless the expression) was present.

The χ^2^ test was used to investigate whether there is a side (laterality) and/or gender (sexual dimorphism) preference. The odds ratio (OR) and 95% confidence interval (CI) were also calculated. Data were analysed using the software PRISM 9 (GraphPad Software, 9.0, San Diego, CA, USA) at a significance level of *p* ≤ 0.050.

## Results

Dental records from 163 orthodontic patients (82 males and 81 females) were included, in primary molars, a total of 40 um1 (r), 39 um1 (l), 65 um2 (r), 68 um2 (l), 32 lm1 (r), 37 lm1 (l), 59 lm2 (r), and 64 lm2 (l). In permanent molars, a total of 162 UM1 (r), 162 UM1 (l), 160 UM2 (r), 158 UM2 (l), 160 LM1 (r), 160 LM1 (l), 149 LM2 (r), and 143 LM2 (l) were investigated. Their age ranged from 8–14 years, and they were in mixed or permanent dentition.

The prevalence of each trait in UM is presented in the Table 3. The most common cusp was the metacone, in which none of the UM showed the absence of metacone (grade 0). The grade 4/5 of hypocone was highly prevalent in UM1. In Carabelli trait negative expression traits (grades 1–4) were more frequent compared to positive expressions (grades 5–7). The metaconule trait presented low frequency in UM. The parastyle cusp was highly uncommon; only 2 teeth were affected. A sexual dimorphism was observed for the hypocone in UM1, in which only females presented reduced expression of the traits (*p* = 0.041; the whole dataset is available in Supplementary Table 1).

**Table 3:** Traits prevalence in the upper permanent molars Prävalenz von Merkmalen der oberen bleibenden Molaren

Trait/Grade	First molars		Second molars		χ^2^ test			
					(laterality)		(dimorphism)	
	UM1 (r)	UM1 (l)	UM2 (r)	UM2 (l)	UM1	UM2	UM1	UM2
*CARABELLI TRAIT, n *(%)								
0. Smooth surface	86 (54.1)	88 (55.0)	14 (91.2)	147 (93.0)	0.985	0.910	0.637	0.279
1. Vertical groove	17 (10.7)	12 (7.5)	5 (3.1)	4 (2.5)				
2. Pit	6 (3.8)	7 (4.4)	4 (2.5)	1 (0.6)				
3. Small Y‑shaped	14 (8.8)	16 (10.0)	2 (1.3)	3 (1.9)				
4. Large Y‑shaped	7 (4.4)	9 (5.6)	1 (0.6)	1 (0.6)				
5. Small cusp	9 (5.7)	8 (5.0)	1 (0.6)	1 (0.6)				
6. Medium cusp	17 (10.7)	17 (10.6)	1 (0.6)	1 (0.6)				
7. Large free cusp	3 (1.9)	3 (1.9)	–	–				
*METACONE, n *(%)								
2. Faint cuspule	–	–	–	1 (0.7)	0.850	0.713	0.791	0.698
3. Weak cusp	–	–	8 (5.3)	10 (6.8)				
4. Large cusp	20 (12.6)	21 (13.3)	85 (56.3)	82 (55.8)				
5. Very large cusp	139 (87.4)	137 (86.2)	58 (38.4)	54 (36.7)				
*HYPOCONE, n *(%)								
0. Smooth surface	–	–	23 (16.5)	17 (12.7)	0.999	0.818	0.041*	0.578
1/2. Faint ridge and Faint cuspule	1 (0.6)	1 (0.6)	34 (24.5)	35 (26.1)				
3. Small cusp	2 (1.3)	2 (1.3)	52 (37.4)	54 (40.3)				
4/5. Large and very large cusp	157 (98.1)	157 (98.1)	30 (21.6)	28 (20.9)				
*METACONULE, n *(%)								
0. Trait is absent	142 (89.9)	145 (92.4)	116 (87.2)	115 (91.3)	0.950	0.962	0.054	0.617
1. Faint cuspule	6 (3.8)	3 (1.9)	4 (3.1)	4 (3.2)				
2. Trace cuspule	5 (3.2)	4 (2.5)	7 (5.4)	5 (3.9)				
3. Small cuspule	1 (0.6)	1 (0.6)	2 (1.5)	1 (0.8)				
4. Small cusp	1 (0.6)	1 (0.6)	1 (0.75)	1 (0.8)				
5. Medium-sized cusp	3 (1.9)	3 (1.9)	–	–				
*PARASTYLE, n *(%)								
0. Smooth surface	162 (100.0)	162 (100.0)	158 (99.4)	154 (99.4)	–	0.999	–	0.150
4. Large cusp	–	–	1 (0.6)	1 (0.6)				

*U* upper, *M* molar, *1* first, *2* second, *(r)* right, *(l)* left

*Statistical difference (*p* ≤ 0.05)

The prevalence of each trait in LM is presented in Table 4. In LM1 the Y groove pattern was the most commonly observed. The hypoconulid was highly prevalent in LM1. Cusp 6 was an uncommon trait and appeared only in LM1. A similar pattern was observed in cusp 7; however, 4 teeth in LM2 presented the trait. The protostylid (a pit feature) was observed in LM1 and LM2 (LM1r = 10.0%; LM1l = 8.7%; LM2r = 2.1%; and LM2l = 2.1%). Anterior fovea was observed in about half of the teeth. Deflecting wrinkles were highly frequent in LM1. The distal trigonid crest was not a common trait. Males presented more hypoconulids than females, when the analysis is performed grouping grades 1–5 compared with grade 0, males (11.3%) presented statistically more hypoconulid traits than females (2.8%; *p* = 0.019; OR = 4.2, CI 95% = 1.3–14.3). Gender differences were also observed for cusp 7, in which only males presented the trait in LM2 (*p* = 0.050). On the other hand, the distal trigonid crest was more common in females. In the LM1 only females (6.2%) presented the distal trigonid crest (*p* = 0.002). In the LM2 the prevalence of distal trigonid crest was 8.2% in males, but 24.1% in females (*p* = 0.0003; OR = 3.6, CI95% =1.7–7.0; the whole dataset is available in Supplementary Table 2).

**Table 4:** Trait prevalence in the lower permanent molars Prävalenz von Merkmalen der unteren bleibenden Molaren

Trait / Grade	First molars		Second molars		χ^2^ test			
					(laterality)		(dimorphism)	
	LM1 (r)	LM1(l)	LM2 (r)	LM2 (l)	LM1	LM2	LM1	LM2
*GROOVE PATTERN, n (%)*								
Y. Cusps 2 and 3 are in contact	94(74.0)	97(77.6)	38(27.3)	37(27.8)	0.623	0.685	0.247	0.067
+. Cusps 1–4 are in contact	22(17.3)	21(16.8)	57(41.0)	60(45.1)				
X. Cusps 1 and 4 are in contact	11(8.7)	7(5.6)	44(31.7)	36(27.1)				
*HYPOCONULID, n (%)*								
0. Absent	22(13.8)	19(11.9)	104(91.2)	89(94.7)	0.994	0.692	0.204	0.070
1. Very small	11(6.9)	13(8.2)	1(0.9)	1(1.1)				
2. Small	34(21.4)	33(20.8)	6(5.3)	2(2.1)				
3. Medium-sized	72(45.3)	74(46.5)	3(2.6)	2(2.1)				
4. Large	17(10.7)	17(10.7)	–	–				
5. Very large	3(1.9)	3(1.9)	–	–				
*CUSP 6, n (%)*								
0. Absent	150(94.3)	151(94.9)	107(100.0)	87(100.0)	0.908	–	0.168	–
1. Much smaller than cusp 5	4(2.5)	3(1.9)	–	–				
2. Smaller than cusp 5	3(1.9)	2(1.3)	–	–				
3. Equal in size to cusp 5	2(1.3)	3(1.9)	–	–				
*CUSP 7, n (%)*								
0. Absent	155(96.9)	157(98.1)	147(98.7)	141(98.6)	0.733	0.967	0.091	0.050*
1. Faint cusp	1(0.6)	1(0.6)	2(1.3)	2(1.4)				
2. Small	1(0.6)	–	–	–				
3. Medium-sized	2(1.3)	2(1.3)	–	–				
4. Large	1(0.6)	–	–	–				
*PROTOSTYLID, n (%)*								
0. Smooth surface	144(90.0)	146(91.3)	141(97.9)	143(97.9)	0.701	0.986	0.701	0.999
1. Pit present	16(10.0)	14(8.7)	3(2.1)	3(2.1)				
*ANTERIOR FOVEA, n (%)*								
0. Absent	77(55.4)	76(53.1)	66(46.1)	56(39.4)	0.983	0.708	0.591	0.184
1. Trace with a weak ridge	14(10.1)	15(10.5)	18(12.6)	19(13.4)				
2. Essential ridges on trigonid developed	43(30.9)	47(32.9)	55(38.5)	63(44.4)				
3. Essential ridges pronounced	5(3.6)	5(3.5)	4(2.8)	4(2.8)				
*DEFLECTING WRINKLE, n (%)*								
0. Absent	45(33.6)	44(31.9)	111(77.1)	111(77.6)	0.871	0.795	0.437	0.932
1. Midpoint constriction	45(33.6)	49(35.5)	19(13.2)	21(14.7)				
2. Deflected distally	37(27.6)	35(25.4)	14(9.7)	11(7.7)				
3. L-shaped ridge	7(5.2)	10(7.2)	–	–				
*DISTAL TRIGONID CREST, n (%)*								
0. Absent	130(97.0)	132(97.1)	123(87.2)	113(81.3)	0.983	0.172	0.002*	0.0003*
1. Present	4(2.9)	4(2.9)	18(12.8)	26(18.7)				

*L* lower, *M* molar, *1* first, *2* second, *(r)* right, *(l)* left

*Statistical difference (*p* ≤ 0.05)

The prevalence of each trait for upper primary molars (um) is presented in Table 5. The Carabelli trait was observed only in um2. The most common crown pattern in um1 was the protocone and paracone; and large hypocone in um2. Metaconule cusp was a rare found, and parastyle trait was absent in the sample. There was no gender or side preference observed in primary molars (the whole dataset is available in Supplementary Table 3).

**Table 5:** Trait prevalence in the upper primary molars Merkmalsprävalenz der oberen Milchmolaren

Trait/Grade	First molars		Second molars		χ^2^ test			
					(laterality)		(dimorphism)	
	um1 (r)	um1 (l)	um2 (r)	um2 (l)	um1	um2	um1	um2
*CARABELLI TRAIT, n *(%)								
0. Smooth mesiolingual surface	40(100.0)	39(100.0)	18(28.6)	21(31.8)	–	0.957	–	0.383
1. Pit, groove	–	–	26(41.3)	24(36.4)				
2. Two grooves are parallel	–	–	6(9.5)	7(10.6)				
3. The area between grooves raised	–	–	8(12.7)	10(15.1)				
4. Free apex	–	–	5(7.9)	4(6.1)				
*CROWN PATTERN (first molars), n (%)*								
2. Protocone and paracone	28(73.7)	27(77.1)	–	–	0.905	–	0.133	–
3M. Protocone, paracone and metacone	3(7.9)	3(8.6)	–	–				
3H. Protocone, paracone and hypocone	3(7.9)	3(8.6)	–	–				
4−. Four cusps present but hypocone reduced	4(10.5)	2(5.7)	–	–				
*CROWN PATTERN (second molars), n (%)*								
3. The distal marginal ridge has a groove	–	–	7(10.9)	8(12.1)	–	0.976	–	0.469
4−. Distal marginal ridge without interruption	–	–	15(23.4)	15(22.7)				
4. Large hypocone	–	–	42(65.2)	43(65.1)				
*METACONULE, n *(%)								
0. Absent	38(100.0)	35(100.0)	61(98.4)	64(98.5)	–	0.973	–	0.227
1. Present	–	–	1(1.6)	1(1.5)				
*PARASTYLE, n *(%)								
0. Absent	40(100.0)	39(100.0)	65(100.0)	68(100.0)	–	–	–	–

*u* upper, *m* molar, *1* first, *2* second, *(r)* right, *(l)* left

The prevalence of each trait for primary lower molar (lm) is presented in Table 6. The most common groove pattern was Y (in which cusps 2 and 3 are in contact). Hypoconulid appeared in all lm2. Cusp 6 and cusp 7 were uncommon traits and were observed only in lm2. Protostylid was an uncommon trait in lm1, but a common trait in lm2. The presence of the different expressions of anterior fovea was prevalent in lm2. The central ridge metaconid showed that this trait described as the cusp 2 ridge is similar to the other cusp is more prevalent in lm1 and lm2. The distal trigonid crest was an uncommon trait in lm1 and lm2. Gender difference was present for groove pattern, in which males presented more Y grade and females more + grade in lm2 (*p* = 0.039). Side preferences were not observed (the whole dataset is available in Supplementary Table 4).

**Table 6:** Trait prevalence in the lower primary molars Merkmalsprävalenz der unteren Milchmolaren

Trait/Grade	First molars		Second molars		χ^2^ test			
					(laterality)		(dimorphism)	
	lm1 (r)	lm1(l)	lm2 (r)	lm2 (l)	lm1	lm2	lm1	lm2
*GROOVE PATTERN, n *(%)								
Y. Cusps 2 and 3 are in contact	20 (95.2)	21 (95.5)	28 (77.8)	34 (89.5)	0.973	0.290	0.701	0.039
+. Cusps 1–4 are in contact	–	–	4 (11.1)	3 (7.9)				
X. Cusps 1 and 4 are in contact	1 (4.8)	1 (4.5)	4 (11.1)	1 (2.6)				
*HYPOCONULID, n *(%)								
0. Absent	25 (89.3)	29 (85.3)	–	–	0.640	–	0.549	–
1. Present	3 (10.7)	5 (14.7)	57 (100.0)	63(100.0)				
*CUSP 6, n *(%)								
0. Absent	28 (100.0)	34 (100.0)	57 (100.0)	62 (98.4)	–	0.339	–	0.275
2. Cusp 6 smaller than cusp 5	–	–	–	1 (1.6)				
*CUSP 7, n *(%)								
0. Absent	29 (100.0)	35 (100.0)	48 (84.2)	53 (82.8)	–	0.968	–	0.422
1. Weak short groove of cusp 3 *	–	–	8 (14.0)	10 (15.6)				
2. Small cusp	–	–	1 (1.7)	1 (1.6)				
*PROTOSTYLID, n *(%)								
0. Smooth surface	31 (96.9)	36 (97.3)	29 (49.1)	31 (48.4)	0.917	0.936	0.101	0.089
1. Pit present	1 (3.1)	1 (2.7)	30 (50.9)	33 (51.6)				
*ANTERIOR FOVEA, n *(%)								
0. Absent	24 (100.0)	29 (93.6)	12 (30.0)	7 (16.7)	0.447	0.396	0.603	0.136
1. Trace with a weak ridge	–	1 (3.2)	6 (15.0)	11 (26.2)				
2. Essential ridges on trigonid developed	–	1 (3.2)	21 (52.5)	21 (50.0)				
3. Essential ridges pronounced	–	–	1 (2.5)	2 (4.8)				
4. Well-defined fovea	–	–	–	1 (2.4)				
*CENTRAL RIDGE OF METACONID, n (%)*								
1. Cusp 2 ridge is similar to the other cusps *	24 (92.3)	31(91.2)	40 (88.9)	46 (92.0)	0.875	0.605	0.065	0.934
2. Ridge is well developed and expands to trigonid ***	2 (7.7)	3 (8.8)	5 (11.1)	4 (8.0)				
*DISTAL TRIGONID CREST, n *(%)								
0. Absent	27(93.1)	31 (91.2)	35 (94.6)	35 (92.1)	0.777	0.665	0.087	0.080
1. Distal borders are connected by a ridge	2 (6.9)	3 (8.8)	2 (5.4)	3 (7.9)				

*l* lower, *m* molar, *1* first, *2* second, *(r)* right, and *(l)* left

*Weak short groove on the lingual ridge of cusp 3

**The essential ridge of the cusp 2 is expressed similar in size and prominence as that of the other cusps

***The essential ridge is very well developed in its thickness on cusp 2 and also expands its width in the trigonid basin

Asymmetry was observed in some traits in low frequency, as follows: for Carabelli’s trait in um2 (12.5%), UM1 (21.1%); hypocone in UM2 (17.5%); groove pattern in LM1(4.2%), LM2 (8.4%), lm1 (5.6%), lm2 (3.2%); hypoconulid in LM1 (7.1%); LM2 (57.1%), lm1 (25%); protostylid in lm2 (6.9%); anterior fovea in LM1 (4.7%), LM2 (10.9%), lm2 (0.03%); deflecting wrinkle in LM1 (17.2%); LM2 (11.8%); and central ridge of the metaconid in lm2 (5%). Other traits presented high prevalence of asymmetry: Carabelli’s trait in UM2 (40.0%); metaconule in UM1 (40.0%), UM2 (66.7%), um2 (100%); cusp 6 in LM1 (40.0%), lm2 (100%); cusp 7 in LM1 (85.7%), lm2 (53.8%); protostylid in LM1 (33.3%), LM2 (50%) and distal trigonid crest in LM2 (55.2%). Trait asymmetry was not present in metacone, crown pattern and parastyle in UM2.

## Discussion

The present study investigated the frequency and variability of non-metric permanent and primary molars crown traits in German orthodontic patients. Additionally, the presence of laterality and sexual dimorphism were also investigated. Although several studies have been focusing on specific traits, such as Carabelli’s trait [23], so far, only few studies have been converging many aspects of dental morphology in living humans, and these studies investigated only few populations and ethnicities, such as Venkatesh et al. [18] and Sujitha et al. [19] who evaluated populations from India, Felemban and Manjunatha [17] who examined a sample from Saudi Arabian, and Aguirre et al. [16] who studied a sample from Colombia. Therefore, in the present study, we investigated an orthodontic sample of German children and teenagers in order to access molar morphology in a population with a middle European ancestry.

One important aspect to be emphasized is that dental size and morphology are studied from an interdisciplinary viewpoint, such as anthropology, paleopathology, archeology forensic science, and dentistry, especially orthodontics. To investigate dental traits requires comprehensive knowledge of morphology, comparative anatomy, function and occlusion [4, 24]. Dental anthropology involves the study of the origin and variations of the human dentition, including the identification of structures such as cusp size, number and location of cusps, occlusal pattern, root configuration, number and position of teeth, and individual measurements [12, 16, 25]. In our study, we used similar methods used in dental anthropology research to investigate the frequency, the sexual dimorphism and the left–right symmetry of molar traits in orthodontic patients.

One of the most well-known methods for assessing the morphology of the permanent dentition is the Arizona State University Dental Anthropology System (ASUDAS) [9] that describes the dental root and crown. A tooth is split into two main parts: the root and the crown, and cusp is the singular part constituting the crown in addition to essential lobes and ridges (the NDCT) [4, 16], which plays an important role in the establishment of dental occlusion. In our study, we used ASUDAS to access and describe 13 NDCT of the permanent dentition, including Carabelli’s trait, metacone, hypocone, metaconule, parastyle, groove pattern, hypoconulid, cusp 6, cusp 7, protostylid, anterior fovea, deflecting wrinkle and distal trigonid crest. For primary dentition, different methods have been proposed to classify primary molars. Thus, in addition to ASUDAS [9], we also used other methods to access NDCT in primary molars [8, 21]. The NDCT for primary molars were identified and scored according to ASUDAS [9]; Hanihara method [8] was used for the crown pattern of 1st and 2nd upper molar, cusp 7, central ridge of the metaconid and distal trigonid crest. For Carabelli’s trait, metaconule and hypoconulid classification, the Sciulli method [21] was used, and for parastyle, groove pattern, cusp 6, protostylid and anterior fovea the ASDUDAS system [9] was used. The three studies established systems for classification allowing measurement of minimal and maximal trait expressions and degrees between these two points [22]. It is important to highlight the importance of using the same method of classification for the permanent and primary dentition [19] for some traits observed in both dentitions.

According to Scott and Pilloud [10], over 90% of the published manuscripts on human dental morphology focus on permanent teeth, despite primary teeth offering another perspective on morphological variation. It has been described that primary teeth hold more primitive traits than permanent teeth [4]. Classic studies from the past century stated that teeth are independent of each other in variation and evolution [26]. Butler [26] and Dahlberg [27] published studies describing the role of cusp variations as a field effect. The most distal member on the tooth crown is the most variable element. This gives us an indication of how NDCTs might be associated with the development of malocclusions as recently described by a research team from India [15]. As some NDCTs, for example the size of the hypocone, the hypoconulid, cusp 6 and cusp 7, influence mesiodistal width of teeth and therefore the amount of space they require in the dental arch. As for deciduous molars, their mesiodistal width correlates directly with the amount of space available for the second dentition. But also in the second dentition, the mesiodistal width of molars influences the amount of posterior crowding and is therefore associated with tooth retention. Our study aimed to characterize the frequency of different NDCTs.

Upper molars have 3 major main cusps and one of them is the metacone (cusp 3) [30]. The last major cusp added during primate evolution is the hypocone (cusp 4) [22]. The metaconule (cusp 5) is an occlusal cusp on the distal border [31]. In our study we observed mesial cusps, such as metacone (cusp 3), demonstrating less variation as compared to distal cusps, such as hypocone (cusp 4) and metaconule (cusp 5). Metacone (cusp 3) was prevalent in grades 4 and 5. Yadav et al. [32] showed similar frequencies for these traits in Indians. In the primary dentition, our results showed low prevalence of metacone (cusp 3) in um1 and higher prevalence of the hypocone (cusp 4) in um2. Sujitha et al. [19] also investigated Indians and reported that metacone (cusp 3) had a high frequency in um1. The parastyle is an accessory cusp on the mesiobuccal surface of upper molars and sometimes linked to Bolk’s paramolar tubercle [9]. The frequency of this morphological trait was below 10% [22], and indeed, in our study only one case was observed in UM2.

Lower molar cusp number depends on the presence of cusp 5, or the hypoconulid. Each cusp is named and numbered. The last major cusp added during primate evolution is the hypoconulid (cusp 5) as a distal cusp integrated more closely with the hypoconid (cusp 3) than entoconid (cusp 4) [22]. In the present study, the frequency of five-cusped molars was predominant on LM1 and lm2. Previous studies support the same result in other populations [11, 19, 34]. Cusp 6, or the entoconulid, is a supernumerary cusp positioned on the distal portion and associated with the entoconid (cusp 4) [22]. Similar to that found in Europeans (5–15%) [22] and in agreement with Kirthiga et al. [33], our study showed a frequency of 5% on LM1. Primary dentition had only one case, which was different from the previous studies that showed a frequency higher than 5% [19, 33]; this difference may be explained by the population difference or by the sample size that was small for primary teeth in our study.

A second supernumerary cusp of the lower molar is cusp 7, or metaconulid, expressed between metaconid (cusps 2) and entoconid (cusp 4). Cusp 7 is relatively rare ranging from 3 to 8% worldwide [22], which is in agreement with the prevalence observed in our study. In contrast to the permanent dentition and similar to previously studies, the frequency on lm2 was higher ranging from 17.2% to 15.8% [11, 16]. Different methods to classify the permanent and primary dentition were used [8, 9], although both considered similar features from cusp 7.

How these NDCTs impact mesiodistal tooth width exactly and how they are connected to malocclusions as crowding or the amount of space available for the eruption of the second dentition is an important question and shall be assessed in future studies. Some NDCTs as Carabelli’s trait or protostylid trait, are not directly connected to the mesiodistal width of the tooth but are also associated with tooth size. This is of interest to the orthodontist, as orthodontic bands are used especially on the first molars in the permanent dentition or on the second deciduous molars. The degree to which these features are pronounced can influence the fit of the orthodontic bands, as these are usually aligned to the most common tooth shape without the mentioned NDCTs. It is therefore advisable for orthodontists, if the expression of the traits is especially pronounced, to consider the necessity of an oral attachment and, sometimes, to forgo the placement of a band and bond a bracket instead.

In our sample the Carabelli’s trait was especially frequent in first molars of the permanent dentition. Carabelli’s trait is a singular derivate expressed on the lingual surface of the protocone and researchers assumed for decades that this is a feature of European-descendant dentition; however, this latter trait was identified in other populations [22, 28]. In our sample, the prevalence of Carabelli’s trait was high in UM1. In the primary dentition, Carabelli’s trait is observed only in um2. A recent systematic review and meta-analysis from Bhavyaa et al. [23] observed a similar prevalence. For um2 the authors reported an estimated prevalence of 72%; likewise the overall prevalence was 59% for UM1 and 8% for UM2. The subgroup analysis also showed that the European continent reported the highest prevalence of Carabelli’s trait [23]. Interestingly, Neanderthals are characterized by the presence of a larger Carabelli trait [29].

The protostylid trait occurs on the buccal surface of the mesiobuccal cusp or protoconid (cusp 1) and is a cingular derivative as Carabelli’s trait [22]. The present study reported in lm2 negative expression. Díaz et al. [11] indicated a high prevalence of this trait on lm2, whereas Sujitha et al. [19] showed a low prevalence. The protostylid was more common in Australopithecines than in modern humans [40].

While some NDCTs influence tooth size, others affect the occlusal relief of the molars and therefore are likely to have an effect on occlusal interdigitation. These traits are of interest as involvement in the development of malocclusion has also been suggested [15]. It is our task as orthodontists to examine the influence of these characteristics on occlusion and to identify possible disruptive factors in dental occlusion. In the current study, the aim was to determine the frequency of the various characteristics in the first place, but studies are to follow that will investigate the involvement of NDCTs in the development of malocclusions.

The major mesial cusps of the lower molars, protoconid (cusp 1) and metaconid (cusp 2), form the trigonid and they can exhibit connected ridges [22]. When the distal accessory ridges run a direct path along the distal portion of the cusps (protoconid and metaconid) and come in contact at a point close to the central occlusal sulcus, the distal trigonid crest is present [8]. This trait is not common in modern humans and was found by Weidenreich [41] on lm2. The noteworthy reduction in trigonid crest prevalence is one hallmark of the modern human dentition [22]. In the present study, the prevalence was 2.9% (LM1) and 18.7% (LM2) in permanent dentition, and 5.4% (lm2) and 8.8% (lm1) in the primary dentition. Hanihara [8] reported results similar to our study, whereas King, Tongkoom and Wong [35] (in a Chinese population) and Sujitha et al. [19] (in an Indian population) reported higher prevalence (33.6%, 65.25% and 93.06%, respectively).

Deflector wrinkle and central ridge of the metaconid are different terms, but all relate to a common feature on lower molars [8, 9]. This trait is expressed on the occlusal surface of metaconid (cusp 2) and is considered a manifestation of the essential ridge. In most instances, this ridge runs from the cusp tip to the central occlusal fossa. The deflecting wrinkles present a wide result variation of the results ranging from lower and high prevalence on a global level [22]. Our study showed more prevalence of presence degrees on LM1 (66.4 and 68.1%). King, Tongkoom and Wong [35] and Sujitha et al. [19] also evaluated the primary dentition using the NDCT classification for the permanent dentition [9]. In their results, deflecting wrinkle was higher on lm2 (63.0 and 87.4% respectively) differing from our study (5.4 and 7.9%). A possible explanation for these result differences may be the variability in the methods used.

The anterior fovea is a polymorphic trait present in the mesial ridge’s aspect on the protoconid (cusp 1) and metaconid (cusp 2) [6]. Data for this feature have not been tabulated on a world scale because Turner et al. [9] did not consider the anterior fovea as one of Turner’s key 29 traits. Our study had prevalent results from presence of different degrees on lm2, LM1 and LM2. It has been reported that Europeans have higher frequencies of this trait in lower molars [22].

Left–right asymmetry is observed in many conditions, including tooth agenesis and tooth morphological alterations, such as microdontia [36]. Deviation from perfect symmetry expressed by changes in structure, as seen in dental morphology studies, is called fluctuating asymmetry [4, 16, 37]. In our study, the Carabelli trait, metaconule (cusp 5), hypoconulid (cusp 5), cusp 6, cusp 7, protostylid and distal trigonid crest were very often observed to be asymmetrical. However, a statistically significant difference was not observed between left and right teeth, showing no side preference.

The investigation of the difference in morphological characteristics of teeth between men and women corroborates the elucidation of sexual dimorphism. Sexual dimorphism is known to be more significant in areas of the dental crown that have a later development [4], which explains our results that observed a male–female difference only in permanent molars. Dental development is influenced by genes located on X and Y chromosomes. The X chromosome is associated with enamel thickness, while the Y chromosome promotes growth of enamel and dentin thickness [4, 38, 39]. The morphological structure, in terms of size and shape of the cusps, is influenced by the sex chromosomes in the formation of their phenotype, although they may not be equally influenced in the formation of enamel and dentin [39, 42, 43]. In our study, gender difference was observed for hypocone, groove pattern, distal trigonid crest and cusp 7, which suggests that sexual chromosomes are carrying markers for these traits.

## Conclusion

The present study showed the frequency of nonmetric dental crown traits (NDCT) of primary and permanent molars in German orthodontic patients and demonstrated that some traits present sexual dimorphism in the permanent and primary teeth. Knowledge regarding the interplay between dental anatomy, physiology, and occlusion is important for clinical practice and should be explored in future studies.

## Supplementary Information

Supplementary Figures 1–2

Supplementary Tables 1–4
