# Correction to: Frequency and variability of nonmetric dental crown traits of primary and permanent molars in a group of orthodontic patients

**DOI:** 10.1007/s00056-024-00538-x

**Published:** 2024-06-20

**Authors:** Ariane Beatriz Blancato, Eva Paddenberg-Schubert, Peter Proff, Maria Angélica Hueb de Menezes-Oliveira, Svenja Beisel-Memmert, Flares Baratto-Filho, Carsten Lippold, Christian Kirschneck, Erika Calvano Küchler, César Penazzo Lepri

**Affiliations:** 1 Department of Biomaterials, University of Uberaba – UNIUBE, Uberaba, Minas Gerais Brazil; 2 Department of Orthodontics, University Medical Centre of Regensburg, Regensburg, Germany; 3 Department of Orthodontics, University of Bonn, Welschnonnenstr. 17, 53111 Bonn, Germany; 4 School of Dentistry, Tuiuti University from Paraná, Curitiba, Paraná Brazil; 5 Department of Dentistry, Univille – University from the Joinville Region, Joinville, Santa Catarina Brazil; 6 Private Orthodontic Practice, Synagogenstr. 1, Ibbenbüren, Germany

**Correction to:**

**J Orofac Orthop 2024**

10.1007/s00056-024-00532-3

In the original version of this article, the given and family names of Cesar Penazzo Lepri were incorrectly structured. The name was displayed correctly in all versions at the time of publication. The original article has been corrected.

