# Supplementary material for: Frequency and variability of nonmetric dental crown traits of primary and permanent molars in a group of orthodontic patients
Source: J Orofac Orthop. 2024 Jun 6;86(5):298–313. doi: 10.1007/s00056-024-00532-3 (PMC12373683; doi:10.1007/s00056-024-00532-3)

Supplementary Figure. 1. Carabelli trait: 1.um2r grade 0, **2.** um2r grade 2, **3.**um2r grade 4; **Crown pattern first molar: 4.**um1r grade 2, **5.**um1r grade 3M, **6.**um1r grade 4 ; **Crown pattern second molar: 7.**um2r grade 3, **8.**um2r grade 4–, **9.**um2r grade 4; **Metaconule: 10.**um2r grade 0, **11**.um2r grade P; **Parastyle: 12.**um2r grade 0; **Groove pattern: 13.**lm2r grade Y , **14.**lm2r grade X , **15.**lm2r grade + ; **Hypoconulid: 16.**lm1l grade 0, **17.**lm2 grade P; **Cusp 6: 18.**lm2l grade 0, **19.**lm2l grade 3; **Cusp 7: 20.**lm2l grade 0, **21.**lm2l grade 2; **Protostylid: 22.**lm2l grade 0, **23.**lm2l grade 1; **Anterior Fovea: 24.**lm2l grade 1, **25.**lm2l grade 2, **26.**lm2l grade 4; **Central ridge of metaconid: 27**.lm2l grade 1, **28.**lm2l grade 2; **Distal Trigonid Crest: 29.**lm2r grade 0, **30.**lm2r grade 1

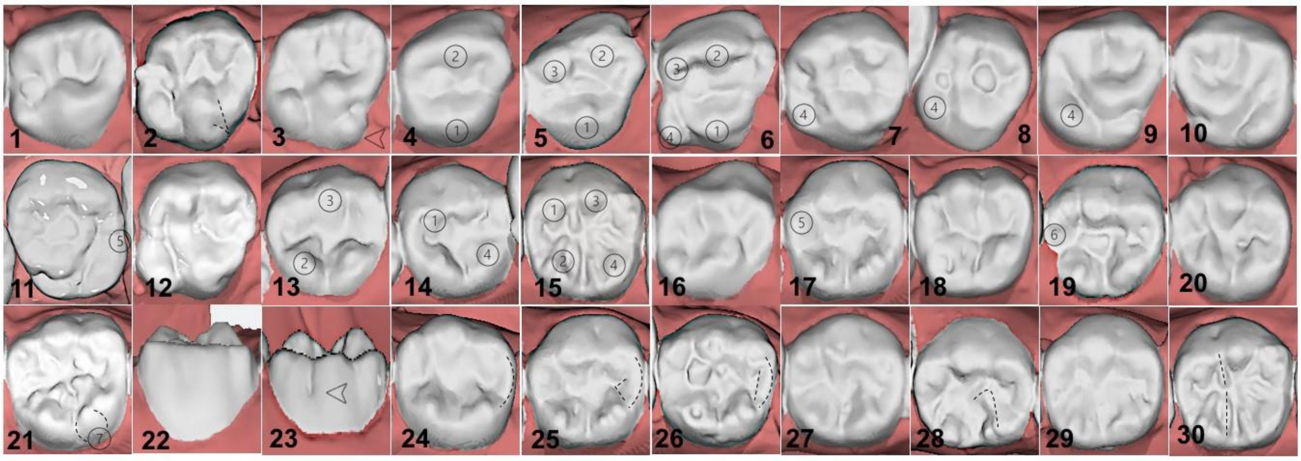

Supplementary Figure 2. Carabelli trait: 1.UM1r grade 0, **2.**UM1r grade 4, **3.**UM1r grade 6; **Metacone: 4.**UM2l grade 2, **5.**UM1l grade 3, **6.**UM1l grade 5; **Hypocone: 7.**UM2r grade 0, **8.**UM1r grade 3, **9.**UM1r grade 5; **Metaconule: 10.**UM1r grade 0, **11**.UM1r grade 4; **Parastyle: 12.**UM1r grade 0, **13.**UM2r grade 5; **Groove pattern: 14.**LM1l grade Y , **15.**LM1l grade X , **16.**LM1l Grade + ; **Hypoconulid: 17.**LM1l grade 0, **18.**LM1l grade 2, **19.**LM1 grade 5; **Cusp 6: 20.**LM1l grade 0, **21.**LM1l grade 2; **Cusp 7: 22.**LM1l grade 0, **23.**LM1l grade 1, **24.**LM1r grade 4; **Protostylid: 25.**LM1l grade 0, **26.**LM1l grade 1; **Anterior Fovea: 27.**LM1l grade 0, **28.**LM1l grade 1, **29.**LM1l grade 4; **Deflecting Wrinkle: 30**.LM2r grade 0, **31.**LM1r grade 2, **32.**LM1r grade 3; **Distal Trigonid Crest: 33.**LM1l grade 0, **34.**LM2l grade 1

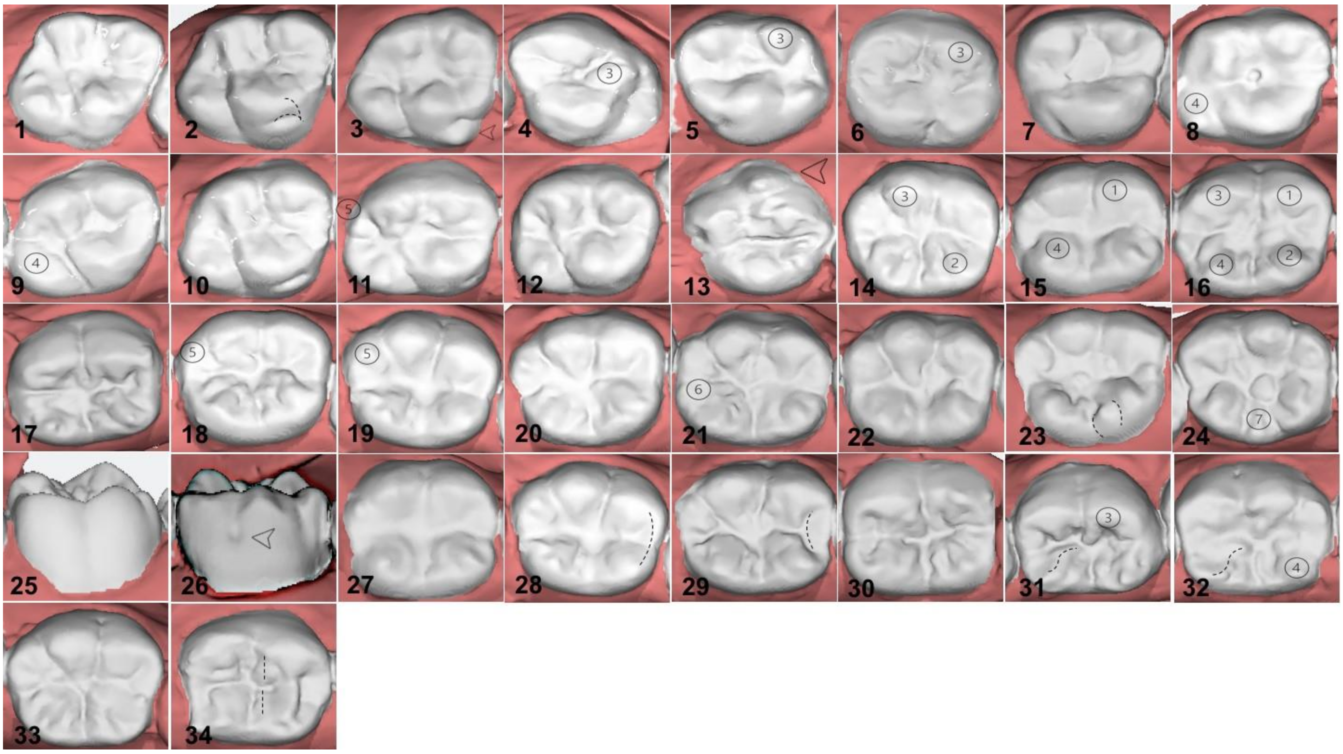

Supplement: Supplementary file 1 — Supplementary Figures 1–2 [file 56_2024_532_MOESM1_ESM.pdf]
