# Supplementary material for: Frequency and variability of nonmetric dental crown traits of primary and permanent molars in a group of orthodontic patients
Source: J Orofac Orthop. 2024 Jun 6;86(5):298–313. doi: 10.1007/s00056-024-00532-3 (PMC12373683; doi:10.1007/s00056-024-00532-3)
Supplement: Supplementary file 2 — Supplementary Tables 1–4 [file 56_2024_532_MOESM2_ESM.pdf]

Supplementary Table 1. Trait prevalence in the upper permanent molars

Trait / Grade	First molars		Second molars		Chi-squared test dimorphism	
	UM1 (m)	UM1 (f)	UM2 (m)	UM2 (f)	UM1	UM2
**CARABELLI TRAIT n(%)**						
0. Smooth surface	85(52.5)	89(56.7)	149(93.1)	144(91.1)	0.637	0.279
1. Vertical groove	12(7.4)	17(10.8)	3(1.9)	6(3.8)		
2. Pit	9(5.6)	4(2.5)	3(1.9)	2(1.3)		
3. Small Y-shaped	16(9.9)	14(8.9)	3(1.9)	2(1.3)		
4. Large Y-shaped	8(4.9)	8(5.1)	-	2(1.3)		
5. Small cusp	10(6.2)	7(4.5)	2(1.2)	-		
6. Medium cusp	20(12.3)	14(8.9)	-	2(1.3)		
7. Large free cusp	2(1.2)	4(2.5)	-	-		
**METACONE n(%)**						
2. Faint cuspule	-	-	1(0.7)	-	0.791	0.698
3. Weak cusp	-	-	8(5.5)	10(6.6)		
4. Large cusp	22(13.4)	19(12.4)	80(54.8)	87(57.2)		
5. Very large cusp	142(86.6)	134(87.6)	57(39.0)	55(36.2)		
**HYPOCONE n(%)**						
0. Smooth surface	-	-	23(16.9)	17(12.4)	0.041*	0.578
1 / 2. Faint ridge and Faint cuspule	-	2(1.3)	36(26.5)	33(24.1)		
3. Small cusp	-	4(2.5)	48(35.3)	58(42.3)		
4 / 5. Large and very large cusp	163(100.0)	151(96.2)	29(21.3)	29(21.2)		
**METACONULE n(%)**						
0. Trait is absent	146(89.6)	141(92.8)	115(90.6)	116(89.9)	0.054	0.617
1. Faint cuspule	3(1.8)	6(3.9)	4(3.1)	4(3.1)		
2. Trace cuspule	4(2.5)	5(3.3)	7(5.5)	5(3.9)		
3. Small cuspule	2(1.2)	-	1(0.8)	2(1.6)		
4. Small cusp	2(1.2)	-	-	2(1.6)		
5 .Medium-sized cusp	6(3.7)	-	-	-		
**PARASTYLE n(%)**						
0. Smooth surface	164(100.0)	160(100.0)	153(98.7)	159(100.0)	-	0.150
4. Large cusp	-	-	2(1.3)	-		

Note: U means upper, M means molar, 1 means first, 2 means second, (m) means male, and (f) means female. *means statistical significant difference ($p \leq 0.05$)

Supplementary Table 2. Trait prevalence in the lower permanent molars

Trait / Grade	First molars		Second molars		Chi-squared test (dimorphism)	
	**LM1 (m)**	**LM1(f)**	**LM2 (m)**	**LM2 (f)**	**LM1**	**LM2**
**GROOVE PATTERN n(%)**						
Y. Cusps 2 and 3 are in contact	94(71.7)	97(80.2)	46(32.6)	29(22.1)	0.247	0.067
+. Cusps 1–4 are in contact	25(19.1)	18(14.9)	52(36.9)	65(49.6)		
X. Cusps 1 and 4 are in contact	12(9.2)	6(4.9)	43(30.5)	37(28.2)		
**HYPOCONULID n(%)**						
0. Absent	24(14.8)	17(10.9)	94(88.7)	99(97.1)	0.204	0.070
1. Very small	7(4.3)	17(10.9)	1(0.9)	1(0.9)		
2. Small	32(19.8)	35(22.4)	6(5.7)	2(1.9)		
3. Medium-sized	75(46.3)	71(45.5)	5(4.7)	-		
4. Large	20(12.3)	14(8.9)	-	-		
5. Very large	4(2.5)	2(1.3)	-	-		
**CUSP 6 n(%)**						
0. Absent	155(95.7)	146(93.6)	101(100.0)	93(100.0)	0.168	-
1. Much smaller than cusp 5	2(1.2)	5(3.2)	-	-		
2. Smaller than cusp 5	4(2.5)	1(0.6)	-	-		
3. Equal in size to cusp 5	1(0.6)	4(2.6)	-	-		
**CUSP 7 n(%)**						
0. Absent	154(95.1)	158(100.0)	146(97.3)	142(100.0)	0.0915	0.050*
1. Faint cusp	2(1.2)	-	4(2.6)	-		
2. Small	1(0.6)	-	-	-		
3. Medium-sized	4(2.5)	-	-	-		
4. Large	1(0.6)	-	-	-		
**PROTOSTYLID n(%)**						
0. Smooth surface	144(90.0)	146(91.3)	142(97.9)	142(97.9)	0.701	0.999
1. Pit present	16(10.0)	14(8.8)	3(2.1)	3(2.1)		
**ANTERIOR FOVEA n(%)**						
0. Absent	79(52.7)	74(56.1)	61(39.9)	61(46.2)	0.591	0.184
1. Trace with a weak ridge	13(8.7)	16(12.1)	23(15.0)	14(10.6)		
2. Essential ridges on trigonid developed	52(34.7)	38(28.8)	67(43.8)	51(38.6)		
3. Essential ridges pronounced	6(4.0)	4(3.0)	2(1.3)	6(4.5)		
**DEFLECTING WRINKLE n(%)**						
0. Absent	51(36.7)	38(28.6)	118(78.1)	104(76.5)	0.437	0.932
1. Midpoint constriction	47(33.8)	47(35.3)	20(13.2)	20(14.7)		
2. Deflected distally	32(23.0)	40(30.1)	13(8.6)	12(8.8)		
3. L-shaped ridge	9(6.5)	8(6.0)	-	-		
**DISTAL TRIGONID CREST n(%)**						
0. Absent	140(100.0)	122(93.8)	135(91.8)	101(75.9)	0.002*	0.0003*
1. Present	-	8(6.2)	12(8.2)	32(24.1)		

Note: L means lower,M means molar, 1 means first, 2 means second, (m) means male, (f) means female. *means statistical significant difference ($p \leq 0.05$)

Supplementary Table 3. Trait prevalence in the upper primary molars

Trait / Grade	First molars		Second molars		Chi-squared test (dimorphism)	
	**um1 (m)**	**um1 (f)**	**um2 (m)**	**um2 (f)**	**um1**	**um2**
**CARABELLI TRAIT n(%)**						
0. Smooth mesiolingual surface	45(100.0)	34(100.0)	21(28.0)	18(33.3)	-	0.383
1. Pit, groove	-	-	30(40.0)	20(37.0)		
2. Two grooves are parallel	-	-	5(6.7)	8(14.8)		
3. The area between grooves raised	-	-	13(17.3)	5(9.3)		
4. Free apex	-	-	6(8.0)	3(5.6)		
**CROWN PATTERN (first molars) n(%)**						
2. Protocone and paracone	33(82.5)	22(66.7)	-	-	0.133	-
3M. Protocone, paracone and metacone	2(5.0)	4(12.1)	-	-		
3H. Protocone, paracone and hypocone	4(10.0)	2(6.1)	-	-		
4–. All four cusps present but hypocone reduced	1(2.5)	5(15.1)	-	-		
**CROWN PATTERN (second molars) n(%)**						
3. The distal marginal ridge has a groove	-	-	8(10.8)	7(12.5)	-	0.469
4–. Distal marginal ridge without interruption	-	-	20(27.0)	10(17.9)		
4. Large hypocone	-	-	46(62.2)	39(69.6)		
**METACONULE n(%)**						
0. Absent	41(100.0)	32(100.0)	72(97.3)	53(100.0)	-	0.227
1. Present	-	-	2(2.7)	-		
**PARASTYLE n(%)**						
0. Absent	45(100.0)	34(100.0)	75(100.0)	58(100.0)	-	-

Note: u means upper, m means molar, 1 means first, 2 means second, (m) means male, (f) means female

Supplementary Table 4. Trait prevalence in the lower primary molars

Trait / Grade	First molars		Second molars		Chi-squared test (dimorphism)	
	**lm1 (m)**	**lm1(f)**	**lm2 (m)**	**lm2 (f)**	**lm1**	**lm2**
**GROOVE PATTERN n(%)**						
Y. Cusps 2 and 3 are in contact	26(96.3)	15(93.8)	37(88.1)	25(78.1)	0.701	0.039
+. Cusps 1–4 are in contact	-	-	1(2.4)	6(18.8)		
X. Cusps 1 and 4 are in contact	1(3.7)	1(6.2)	4(9.5)	1(3.1)		
**HYPOCONULID n(%)**						
0. Absent	33(89.2)	21(84.0)	-	-	0.549	-
1. Present	4(10.8)	4(16.0)	65(100.0)	55(100.0)		
**CUSP 6 n(%)**						
0. Absent	37(100.0)	25(100.0)	65(100.0)	54(98.2)	-	0.275
2. Cusp 6 smaller than cusp 5	-	-	-	1(1.8)		
**CUSP 7 n(%)**						
0. Absent	39(100.0)	25(100.0)	54(81.8)	47(85.5)	-	0.422
1. Weak short groove on the lingual ridge of cusp 3	-	-	10(15.2)	8(14.5)		
2 .Small cusp	-	-	2(3.0)	-		
**PROTOSTYLID n(%)**						
0. Smooth surface	39(100.0)	28(93.3)	28(41.8)	32(57.1)	0.101	0.089
1. Pit present	-	2(6.7)	39(58.2)	24(42.9)		
**ANTERIOR FOVEA n(%)**						
0. Absent	35(94.6)	18(100.0)	14(29.2)	5(14.7)	0.603	0.136
1. Trace with a weak ridge	1(2.7)	-	7(14.6)	10(29.4)		
2. Essential ridges on trigonid developed	1(2.7)	-	23(47.9)	19(55.9)		
3. Essential ridges pronounced	-	-	3(6.2)	-		
4 .Well-defined fovea	-	-	1(2.1)	-		
**CENTRAL RIDGE OF METACONID n(%)**						
1. Cusp 2 ridge is similar to the other cusps *	32(86.5)	23(100.0)	49(90.7)	37(90.2)	0.065	0.934
2. Ridge is well developed and expands to trigonid **	5(13.5)	-	5(9.3)	4(9.8)		
**DISTAL TRIGONID CREST n(%)**						
0. Absent	36(87.8)	22(100.0)	42(97.7)	28(87.5)	0.087	0.080
1.Distal borders are connected by a ridge	5(12.2)	-	1(2.3)	4(12.5)		

Note: l means lower, m means molar, 1 means first, 2 means second, (m) means male, (f) means female
* The essential ridge of the cusp 2 is expressed similar in size and prominence as that of the other cusps; ** The essential ridge is very well developed in its thickness on cusp 2 and also expands its width in the trigonid basin
